# Functions of NQO1 in Cellular Protection and CoQ_10_ Metabolism and its Potential Role as a Redox Sensitive Molecular Switch

**DOI:** 10.3389/fphys.2017.00595

**Published:** 2017-08-24

**Authors:** David Ross, David Siegel

**Affiliations:** Department of Pharmaceutical Sciences, Skaggs School of Pharmacy, University of Colorado Anschutz Medical Campus Aurora, CO, United States

**Keywords:** quinone reductases, quinones, coenzyme Q, vitamin E, superoxide, polymorphism, single nucleotide

## Abstract

NQO1 is one of the two major quinone reductases in mammalian systems. It is highly inducible and plays multiple roles in cellular adaptation to stress. A prevalent polymorphic form of NQO1 results in an absence of NQO1 protein and activity so it is important to elucidate the specific cellular functions of NQO1. Established roles of NQO1 include its ability to prevent certain quinones from one electron redox cycling but its role in quinone detoxification is dependent on the redox stability of the hydroquinone generated by two-electron reduction. Other documented roles of NQO1 include its ability to function as a component of the plasma membrane redox system generating antioxidant forms of ubiquinone and vitamin E and at high levels, as a direct superoxide reductase. Emerging roles of NQO1 include its function as an efficient intracellular generator of NAD^+^ for enzymes including PARP and sirtuins which has gained particular attention with respect to metabolic syndrome. NQO1 interacts with a growing list of proteins, including intrinsically disordered proteins, protecting them from 20S proteasomal degradation. The interactions of NQO1 also extend to mRNA. Recent identification of NQO1 as a mRNA binding protein have been investigated in more detail using SERPIN1A1 (which encodes the serine protease inhibitor α-1-antitrypsin) as a target mRNA and indicate a role of NQO1 in control of translation of α-1-antitrypsin, an important modulator of COPD and obesity related metabolic syndrome. NQO1 undergoes structural changes and alterations in its ability to bind other proteins as a result of the cellular reduced/oxidized pyridine nucleotide ratio. This suggests NQO1 may act as a cellular redox switch potentially altering its interactions with other proteins and mRNA as a result of the prevailing redox environment.

## Introduction

There are many one- and two-electron reductases in cellular systems capable of reduction of quinones to semiquinones and hydroquinones respectively. NQO1 and NQO2 are the two mammalian forms of the obligate two-electron reductase family termed NAD(P)H:quinone acceptor oxidoreductases (NQO). The role of NQO1 in chemoprotection has been extensively reviewed (Ernster, 1967; De Long et al., 1986; Lind et al., 1990; Cadenas et al., 1992; Cadenas, 1995; Ross et al., 2000) and there is detailed information available regarding NQO1 structure and mechanism (Hosoda et al., 1974; Li et al., 1995; Tedeschi et al., 1995; Bianchet et al., 2004). Crystal structures of both NQO1 and NQO2 have been published (Foster et al., 1999; Skelly et al., 1999; Faig et al., 2000) allowing structural and functional comparisons between NQO1 and NQO2 (Jaiswal et al., 1990; Zhao et al., 1997; Bianchet et al., 2004). In this article we will focus on NQO1, its functions, polymorphisms and potential relevance to ubiquinone mediated antioxidant protection. In addition, we will summarize data which points to a potential role for the redox environment in controlling NQO1 structure and downstream functions.

## Induction of NQO1

NQO1 is a highly inducible protein under a variety of stress responses including oxidative stress (Prochaska et al., 1992; Joseph et al., 1994). Induction of the protein to high levels in cells can be mediated by Nrf2-mediated induction or via Ah receptor mechanisms (Vasiliou et al., 1994; Jaiswal, 2000). The key point to be emphasized is that NQO1 levels in cells can increase rapidly under stress conditions presumably as a cellular protective system (see below) or an as yet to be identified component of a more generalized cellular adaptive response.

## Functions of NQO1

### Quinone reduction

**Figure 1:**
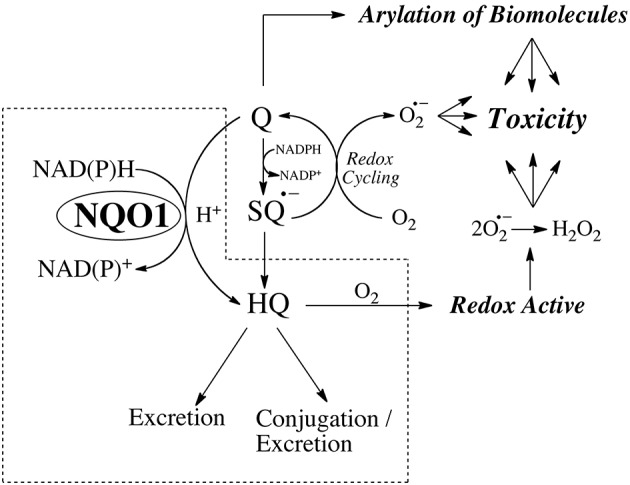
The properties of the hydroquinone determine whether reduction by NQO1 leads to detoxification or toxicity.

NQO1 is extremely effective at catalyzing the two-electron mediated reduction of quinones to hydroquinones (Ernster, 1967; Hosoda et al., 1974; Lind et al., 1982; Thor et al., 1982) which is commonly proposed as a mechanism of detoxification. Quinones, dependent on structure, are electrophilic species capable of reaction with cellular nucleophiles and two-electron mediated reduction removes a reactive electrophile from a biological system. In addition, two-electron reduction bypasses the formation of semiquinones which, dependent on redox potential, can interact with molecular oxygen to generate aggressive oxygen and nitrogen species capable of inducing cellular damage. The major caveat to this interpretation is the stability of the hydroquinone that is generated via NQO1 mediated reduction. As indicated in Figure 1, if the hydroquinone is redox active (e.g., β-lapachone) or capable of rearrangement to a reactive alkylating species (e.g., mitomycin C, RH1) then NQO1 mediated bioreduction will represent a *bioactivatio*n rather than a detoxification step and this overall concept has been termed bioreductive activation (Kennedy et al., 1980). A third mechanism of bioactivation as a result of reduction is if the hydroquinone that is generated has greater activity against a particular target when compared to the quinone. This phenomenon has been observed with benzoquinone ansamycin Hsp90 inhibitors where the hydroquinone reduction product has greater affinity for the active site of the Hsp90 ATPase target protein (Guo et al., 2005, 2006; Reigan et al., 2011) when compared to the parent quinone. The ability of hydroquinones with different redox stabilities to generate toxicity was investigated most recently in a study of the effect of quinones on cellular protein handling systems and toxicity where the addition of NQO1 led to opposing effects depending on the stability of the hydroquinone (Xiong et al., 2014). NQO1 generated a relatively stable hydroquinone and protected against 1,4-benzoquinone-induced toxicity while it generated oxygen-labile hydroquinones and potentiated the toxicity of aminochrome (the cyclized quinone formed from dopamine) and menadione with toxicity being closely associated with induction of the ER stress response (Xiong et al., 2014).

## Reduction of ubiquinone and Vitamin E derivatives to antioxidant forms

### Ubiquinone

The reduction of CoQ derivatives of various chain lengths by NQO1 has been examined in both artificial and natural membrane systems (Beyer et al., 1996). NQO1 was able to maintain the reduced form of CoQ_9_ and CoQ_10_ in unilamellar or multilamellar vesicles and protect membrane components from free radical damage and lipid peroxidation. The rate of reduction of CoQ homologs was faster with shorter substituted carbon chain lengths (C_1_, C_2_) vs. longer chain lengths (C_9_, C_10_). This study was extended to examine the effect of NQO1 in mitochondrial membranes of hepatocytes. CoQ_10_ protected against membrane damage after treatment with adriamycin and the protection against loss of membrane permeability could be blocked by inhibitors of NQO1. A concluding suggestion of this work was that NQO1 was selected during evolution as a CoQ reductase and its conversion of xenobiotics and other synthetic molecules was secondary and coincidental to its primary effects on ubiquinone (Beyer et al., 1996).

### Vitamin E

Vitamin E quinone (α-tocopherol quinone, TQ) is a product of free radical attack of Vitamin E and is devoid of direct antioxidant capability (Liebler, 1993). Reduction of TQ to TQ-hydroquinone (TQ-HQ), however, generates a potent antioxidant (Bindoli et al., 1985; Kohar et al., 1995) and can restore antioxidant activity in a biological system. In *in vitro* studies TQ was found to be efficiently reduced by recombinant human NQO1 to TQ-hydroquinone (Siegel et al., 1997). Using a series of Chinese hamster ovary cells stably transfected with varying levels of human NQO1 it could be shown that cells with elevated NQO1 generated and maintained higher levels of TQ-hydroquinone. Cells generating higher levels of TQ-HQ were better protected against cumene hydroperoxide-induced lipid peroxidation (Siegel et al., 1997). The suggestion was made that one of the physiological functions of NQO1 was to generate antioxidant forms of Vitamin E and maintain the antioxidant capability of an oxidized vitamin E molecule in a biological system (Siegel et al., 1997).

## NQO1 as a superoxide reductase

The contribution of NQO1 to antioxidant protection may also be more direct rather than being mediated by reduced derivatives of ubiquinone and vitamin E. NQO1 is a flavoprotein and the flavin co-factor plays a role in the direct scavenging of superoxide with the enzyme functioning as a superoxide reductase (Siegel et al., 2004; Zhu et al., 2007). These reactions have also been described for free flavins (King et al., 1973; Muller, 1987). Experimental studies confirmed the catalytic role of NQO1 as a superoxide reductase using a wide variety of superoxide generating systems and directly using EPR spectroscopy (Siegel et al., 2004; Zhu et al., 2007). A potential direct superoxide scavenging activity for NQO1 raises the question of whether this process is relevant in cellular systems. Cells possess numerous mechanisms to modulate superoxide levels and the superoxide dismutase family (SOD) is an extremely efficient enzyme system which removes superoxide generating hydrogen peroxide (McCord and Fridovich, 1969). The rate of reaction of NQO1 with superoxide is less than an order of magnitude higher than chemical dismutation of superoxide and at least four orders of magnitude less than the rate of enzymatic dismutation of superoxide by SOD. These rate comparisons would argue that the reaction of NQO1 with superoxide has little relevance in cells but an important point is that NQO1 is expressed at relatively high levels under basal conditions in many cell types as well as being highly inducible via Nrf2 and Ah receptor dependent systems. Evidence for the induction of NQO1 by oxidative stress is observed following x-ray and UV radiation, which generate oxidative stress, and can induce NQO1 (up to 30-fold) in human cells (Boothman et al., 1993). We approached the question of the potential relevance of NQO1 in superoxide scavenging in cells using an NQO1 transfected series of Chinese hamster ovary cells (Siegel et al., 2004). Expression of high levels of NQO1 in Chinese hamster ovary cells resulted in increased scavenging of superoxide suggesting that this mechanism may have cellular relevance. It is also an attractive mechanism to potentially ameliorate the oxidative stress induced via unstable hydroquinones generated by NQO1 at their site of generation. It is important to emphasize that any role of NQO1 as a superoxide reductase in the absence of high levels of enzyme is likely to be minimal.

### The plasma membrane redox system

**Figure 2:**
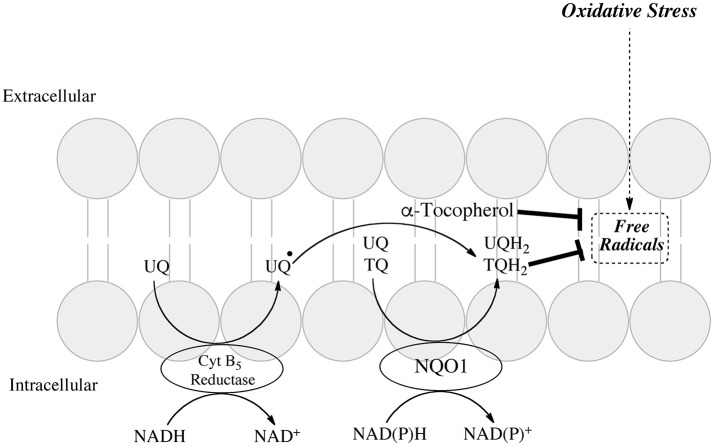
Role of NQO1 in generating antioxidant forms of ubiquinone and α-tocopherol in the plasma membrane redox system (adapted from Hyun et al., 2006).

The plasma membrane redox system (PMRS) is an important component of the cell's ability to defend itself against oxidative stress. The system comprises antioxidants, enzymatic and chemical reductants, and a source of reducing equivalents usually NADPH (Navas et al., 2005; Hyun et al., 2006). Major antioxidant systems contained in the plasma membrane include ubiquinone and vitamin E allowing scavenging of damaging free radicals and inhibition of lipid peroxidation. Reductases contained in the plasma membrane include cytochrome b5 reductase, NQO1 and an additional cytosolic NADPH CoQ reductase (Takahashi et al., 1992). A schematic of the PMRS adapted from (Hyun et al., 2006) is shown in Figure 2. The enzymatic reductases maintain ubiquinone or vitamin E quinone in their hydroquinone forms. Ubiquinol can provide antioxidant protection either alone or in combination with vitamin E or ascorbate (Crane, 2001).

## NQO1 as an NAD^+^ generating system: studies with β-lapachone

**Figure 3:**
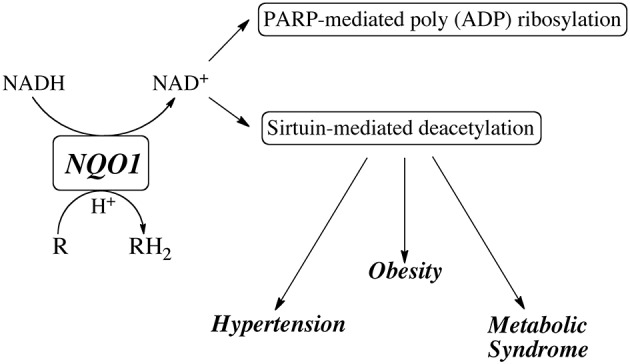
Potential role for NQO1 in generating NAD^+^ for utilization by sirtuins and PARP.

In recent years, attention has been focused on using NQO1 to modulate the NAD^+^/NADH redox balance primarily through the metabolism of the redox-cycling quinone β-lapachone. NQO1 efficiently reduces β-lapachone to the hydroquinone which rapidly autoxidized back to the quinone while consuming reduced pyridine nucleotides (NADH and NADPH) and generating oxidized pyridine nucleotides (NAD^+^ and NADP^+^) (Pink et al., 2000). Treatment with β-lapachone has been shown to modulate a number of physiological processes particularly those involved in the metabolic syndrome including reduction in spontaneous hypertension (Kim et al., 2011, 2013, 2015; Kim Y. H. et al., 2014), amelioration of obesity (Hwang et al., 2009) and reduction in inflammation (Byun et al., 2013; Lee et al., 2015). β-lapachone has also been reported to prevent health declines in aged mice (Lee et al., 2012) and inhibit restenosis by suppressing vascular smooth muscle cell proliferation (Kim et al., 2009). A common theme in many of these studies was the ability of β-lapachone to activate the AMP-activated protein kinase (AMPK) pathway. In addition, treatment with β-lapachone was also shown to reduce cisplatin-mediated nephrotoxicity (Gang et al., 2013; Oh et al., 2014) and hearing loss (Kim H. J. et al., 2014), as well as afford protection to the kidney from salt-induced and ischemia-reperfusin injury (Kim et al., 2012; Gang et al., 2014). In these studies β-lapachone was shown to influence the activities of many pathways including the sirtuins, poly ADP ribose polymerase (PARP) and NAD(P)H oxidase (NOX). These studies suggest that modulation of the NAD(P)^+^/NAD(P)H redox balance by NQO1-mediated metabolism of β-lapachone or other redox active quinones may have therapeutic potential via the ability to generate NAD^+^ and stimulate sirtuin and PARP activities (Figure 3). These data also suggest that individuals with lower NQO1 catalytic activity due to the expression of the NQO1^*^2 mutant allele may be compromised in their ability to influence the NAD(P)^+^/NAD(P)H redox balance after quinone exposure or other stress-related events.

## “Non metabolic” functions of NQO1

### Protein binding

A potentially important role of NQO1 is its ability to physically interact with other proteins and there are numerous examples where such interactions are relevant for cellular function. NQO1 associates with the tumor suppressor p53 in a protein-protein interaction and NQO1 has been reported to stabilize p53 protecting it against ubiquitin-independent 20S proteasomal degradation (Asher et al., 2001, 2002a,b). This provides a potential explanation for the lower basal levels of p53 observed in NQO1-null mice (Iskander et al., 2005). NQO1 has been shown to stabilize a growing number of proteins including p53, p63 p73, ornithine decarboxylase and PGC-1α (Asher et al., 2001, 2002b, 2005a; Hershkovitz Rokah et al., 2010; Adamovich et al., 2013) in a pyridine nucleotide dependent manner. The diversity of proteins which interact with NQO1 has grown significantly and recent work demonstrated stabilization of HIF-1α by NQO1 in RKO colon tumors in mice xenografts as a result of impaired proteasomal degradation (Oh et al., 2016). In a comprehensive study, these authors demonstrated that overexpression of NQO1 in RKO colon tumor xenografts led to significantly increased tumor growth rate and that knockdown of NQO1 inhibited growth but only in the context of functional HIF-1α. This suggested that the increased growth rate was due to stabilization of HIF-1α by NQO1 (Oh et al., 2016). Many of the NQO1 interacting proteins have intrinsically disordered regions and such proteins are often degraded by the 20S proteasome since they require minimal unfolding (Asher and Shaul, 2006; Moscovitz et al., 2012).

In addition to interactions with proteins undergoing degradation, there is also evidence for direct interaction of NQO1 with the 20S proteasome. In yeast, Lot6, the human ortholog of NQO1 binds to the 20S proteasome and recruitment of a transcription factor, Yap4, to the complex results in Yap4 stabilization (Sollner et al., 2009). In mouse liver, NQO1 is found in association with the 20S proteasome using both co-fractionation techniques and co-immunoprecipitation (Asher and Shaul, 2006). Using mass spectrometry, purified NQO1 was found to interact with purified 20S proteasome in a cell free system (Moscovitz et al., 2012). These experiments suggest a modulatory role for NQO1 at the level of the 20S proteasome. However, regulation of the 20S proteasome is complex (Ben-Nissan and Sharon, 2014) and the precise molecular mechanisms of how NQO1 inhibits protein degradation remain to be elucidated.

## RNA binding and control of translation

**Figure 4:**
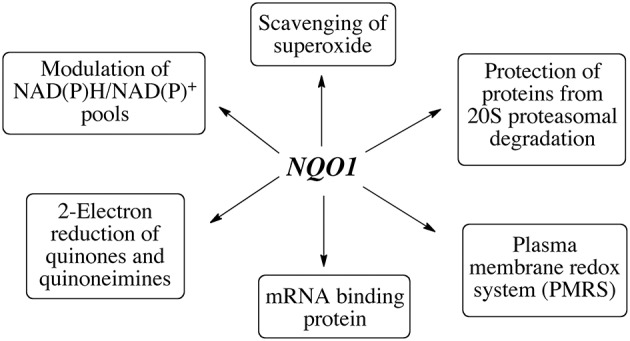
The diverse functions of NQO1.

In a study designed to capture proteins bound to mRNA, a number of FAD containing oxidoreductases including NQO1 were identified as mRNA binding proteins (Castello et al., 2012). A recent study employing ribonucleoprotein immunoprecipitation has shown that NQO1 binds a subset of mRNA's in HepG2 cells and a major target was SERPIN 1A1 mRNA which encodes the serine protease inhibitor α-1-antitrypsin, A1AT, which is associated with disorders including obesity-related metabolic inflammation and chronic obstructive pulmonary disease (COPD), liver cirrhosis and hepatocellular carcinoma (Di Francesco et al., 2016). Biotin pulldown analysis and luciferase reporter analysis indicated that NQO1 could bind the 3” untranslated region and increase SEPIN1A1 translation. It is still too early to determine the potential importance of the mRNA binding of NQO1 but further studies to determine the specificity of binding and whether NQO1 can play a broader role in regulating translation are warranted. In summary, NQO1 has multiple functions in the cell which are summarized in Figure 4.

## NQO1 polymorphisms

Over 20 SNPs have been discovered in NQO1 (Nebert et al., 2002). The most studied NQO1 SNP is the NQO1^*^2 mutant allele characterized as a C to T base-pair substitution at position 609 of the human cDNA resulting in a proline to serine change at amino acid position 187 (Traver et al., 1992, 1997). The mutant NQO1^*^2 protein is rapidly ubiquitinated (Tsvetkov et al., 2011) and degraded by the proteasomal system resulting in lower levels of NQO1 protein in individuals heterozygous for the NQO1^*^2 allele while individuals homozygous for the NQO1^*^2 allele have no NQO1 activity and only trace to non-detectable levels of NQO1 protein (Siegel et al., 1999, 2001). The frequency of homozygous mutant alleles (NQO1^*^2/^*^2) varies widely among different ethnic populations and has been shown to range from 4% in Caucasian to as high as 34% in the Hmong population of South East Asia (Gaedigk et al., 1998; Kiffmeyer et al., 2004). As a result of this amino acid substitution the mutant NQO1^*^2 protein displays a decreased affinity for the FAD cofactor resulting in destabilization of the NQO1 protein (Chen et al., 1999; Pey et al., 2014). The mechanisms underlying the instability of the NQO1^*^2 mutant protein have been the subject of recent work. Lienhart et al. showed that surprisingly, the crystal structures of the P187S mutant and wild type forms of NQO1 were very similar and utilized NMR and limited proteolysis to explore structural differences (Lienhart et al., 2014). These experiments revealed that the single amino acid change destabilized interactions between the core and the C-terminus leading to a less competent FAD binding pocket. Pey et al. showed that the P187S form had a lower binding affinity for the FAD co-factor and addition of FAD could restore stability of the mutant form to wild type levels (Pey et al., 2014). The P187S change destabilizes the NQO1 dimer and increases the flexibility of the C terminal domain (Pey et al., 2016). Recent work has shown that the P187S change causes structural and dynamic changes far from the mutated site via an allosteric mechanism affecting FAD binding at the N-terminal domain and accelerating proteasomal degradation through dynamic effects at the C-terminal domain. These studies argue that FAD binding and the stability of the C-terminal domain are important mechanisms in the accelerated proteasomal degradation of the NQO1 ^*^2 mutant protein. The ubiquitin ligase CHIP recognizes FAD deficient enzymes such as the NQO1 ^*^2 protein and the C-terminal region contributes to recognition by CHIP and subsequent degradation by the proteasome (Martinez-Limon et al., 2016). Importantly, the same inherent instability of FAD-deficient *wild type* NQO1 to proteasomal degradation was observed confirming previous work demonstrating a critical role for the FAD cofactor in maintaining NQO1 stability (Chen et al., 1994; Moscovitz et al., 2012). Martinez –Limon et al. also showed that a lack of FAD caused by precursor vitamin B2 deficiency led to a more general destabilization of flavoproteins even when they had wild type sequences while the general proteome was not affected (Martinez-Limon et al., 2016). In general, the FAD-free, apo forms of flavoproteins were found to be unstable and undergo degradation. Given the importance and extent of the human flavoproteome (Lienhart et al., 2013), FAD levels and incorporation of FAD into precursor proteins to generate mature forms are likely to be critical in cellular protein quality control.

A second polymorphism in NQO1 has also been characterized as NQO1^*^3 (Pan et al., 1995, 2002; Hu et al., 1996) and is a C to T base-pair substitution at position 465. This mutation disrupts the consensus sequence at the 5′-splice site, which is required for binding of U1 small nuclear RNA in spliceosomes resulting in a lower level of NQO1 protein expression (Pan et al., 1995). The NQO1^*^3 polymorphism is much less frequent in human populations and modestly reduces NQO1 enzymatic activity and protein levels (Gaedigk et al., 1998; Pan et al., 2002; Pey et al., 2014) and consequently, may have less profound implications for phenotype than the NQO1^*^2 polymorphism. A recent study examined the mutant NQO1^*^3 protein using biochemical and structural analysis (X-ray crystallography, 2D-NMR) and concluded that the NQO1^*^3 variant is very similar to the wildtype NQO1^*^1 protein and that there were no obvious variations to explain the observed decreased activity (Lienhart et al., 2017).

## Alternatively spliced forms of NQO1

Initial characterization of the NQO1 gene (6 exons and 5 introns) by Jaiswal *et al*. described 3 mRNA species (Jaiswal et al., 1988). Gasdaska et al. confirmed that the NQO1 message undergoes alternative splicing and identified and characterized a variant that was missing exon 4 (Gasdaska et al., 1995). This variant transcript was found in both normal and tumor tissues, although the corresponding protein could not be detected and recombinant NQO1 protein missing exon 4 was shown to have very low catalytic activity toward quinone substrates (Gasdaska et al., 1995). Interestingly, it was later shown by Pan et al. that colon cancer cells carrying the NQO1^*^3 polymorphism (465C > T) had higher levels of NQO1 exon 4 deleted transcript. This was due to defective binding of U1 small nuclear RNAs to the splicesome as a result of the influence of the NQO1^*^3 polymorphism on the consensus sequence at the 5'-splice site (Pan et al., 2002). More recently, seven NQO1 variant transcripts have been identified of which six code for potential NQO1 isoforms (Yates et al., 2016). The higher levels of exon deleted variants of NQO1 represents a potential explanation for the decreased enzymatic activity of cells carrying the NQO1^*^3 polymorphism (Lienhart et al., 2017).

## Post-translational modifications of NQO1

Post-translational modifications (PTM) of NQO1 including phosphorylation, ubiqitination and acetylation have been detected in large proteomic studies. These data are available at the online resource PhosphoSitePlus (http://www.phosphosite.org) and show that PTMs have been detected in many regions of the NQO1 protein. The functions, however, of the post-translationally modified forms of NQO1 remain to be elucidated.

## NQO1 polymorphisms affecting ubiquinone metabolism

There is little information available regarding NQO1 polymorphisms affecting ubiquinone and ubiquinol levels and a comprehensive study is warranted. However, a preliminary study suggested that there may be some influence of the NQO1^*^2 polymorphism on ubiquinone metabolism (Fischer et al., 2011). Plasma levels of CoQ_10_ were lower in individuals heterozygous for the NQO1^*^2 polymorphism than homozygotes (Fischer et al., 2011). However since the population under study was primarily Caucasian and contained only two individuals homozygous for the NQO1^*^2 population, a larger study would be justified in Asian populations where the prevalence of the NQO1^*^2 population is markedly higher.

## NQO1 as a redox sensitive molecular switch

**Figure 5:**
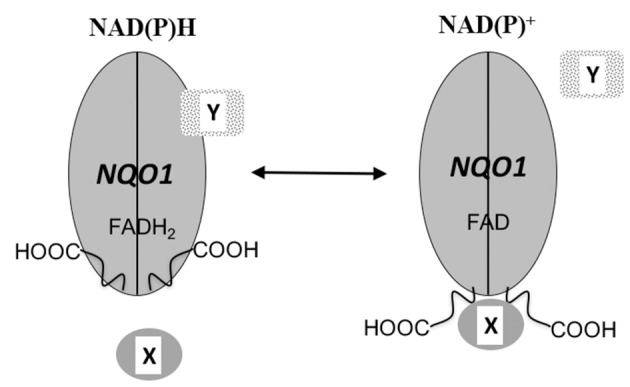
NQO1 as a redox switch. A schematic diagram of NQO1 in the role of a redox responsive molecular switch. The conformation of NQO1 changes in response to levels of reduced pyridine nucleotides. Under normal condition adequate levels of NAD(P)H keep NQO1 in the reduced conformation (FADH_2_) but when NAD(P)H levels fall, NQO1 adopts an oxidized conformation (FAD) exposing its C-terminus domains (-COOH). The change in conformation induced in NQO1 in response to the NAD(P)^+^ / NAD(P)H redox balance alters the binding of either target proteins or RNA to NQO1. X and Y designate NQO1 binding proteins or RNA molecules and it is possible that depending upon the individual target binding to NQO1 may be either increased or reduced by changes in the intracellular NAD(P)^+^ / NAD(P)H ratios.

The crystal structure of NQO1 reveals that the catalytically active form is generated from two interlocking monomers to form a homodimeric protein (Li et al., 1995; Skelly et al., 1999; Asher et al., 2006). The active sites are located at the interface between monomers where they create a non-covalent binding pocket for FAD. Both active sites are identical and each is shared by both reduced pyridine nucleotide and substrate. The catalytic cycle is initiated by the binding of reduced pyridine nucleotide in the active site followed by a hydride transfer to FAD (Hosoda et al., 1974). It is proposed that the enzyme then undergoes a conformational change expelling the oxidized pyridine nucleotide and creating the environment for quinone binding (Hosoda et al., 1974). Due to this *ping-pong-bi-bi* kinetic mechanism NQO1 can exist in either an oxidized (FAD) or reduced (FADH_2_) conformation depending upon the relative concentrations of reduced pyridine nucleotides and substrates. It has been hypothesized that the conformational change in NQO1 that occurs following hydride transfer to FAD is to accommodate the charge separation in the reduced planer form of the flavin (Cavelier and Amzel, 2001). Structural evidence for a conformation change in NQO1 following reduction by reduced pyridine nucleotides has been elusive due to the inability to crystalize purified NQO1 in the reduced state. Biochemical evidence for a conformational change in NQO1 was presented by Chen et al. where they demonstrated that following binding of reduced pyridine nucleotides the C-terminal domains of NQO1 were protected against proteolytic digestion (Chen et al., 1994). The conformational change induced in NQO1 in response to binding of reduced pyridine nucleotides suggests that NQO1 could function as a redox switch where the conformation of the protein can be modulated by the NAD(P)^+^/NAD(P)H redox balance (Figure 5). The ability of NQO1 to protect target proteins from 20S proteolytic cleavage has been shown to be dependent upon the addition of NADH (Asher et al., 2005b; Garate et al., 2008; Hershkovitz Rokah et al., 2010). Similarly, the binding of NQO1 to SERPINA1 mRNA was also shown to be influenced by the NAD(P)^+^/NAD(P)H redox balance (Di Francesco et al., 2016). This suggests that the pyridine nucleotide redox environment controls the interaction of NQO1 with proteins and mRNA and may function as a redox-dependent molecular switch to modulate the downstream cellular functions of NQO1 (Figure 5). Consistent with this overall concept is the finding that in yeast, the FMN-dependent quinone reductase Lot6 has been shown to act as a redox switch which senses oxidative stress and modulates the binding of the transcription factor Yap4 to the 20s proteasome (Sollner et al., 2009). In summary, pyridine nucleotide ratios appear to be critical in NQO1 downstream functions and are likely to modulate the interaction of NQO1 with other signaling proteins.

## Concluding remarks

NQO1 has been shown to play a pivotal role in quinone metabolism generating hydroquinones which possess chemical properties distinct from their parent quinone. The stability of the hydroquinone generated is critical in determining whether NQO1 will detoxify a reactive quinone or lead to enhanced toxicity. While initially classified as a detoxification enzyme the role of NQO1 has expanded beyond quinone metabolism to encompass a broad spectrum of biological functions. Provision of a cellular source of NAD^+^ which may be particularly important in preventing or inhibiting metabolic syndrome, protection of proteins from proteasomal degradation and enhancing protein translation by binding to mRNA represent emerging functions. The role of NQO1 as an important component of the PMRS and its ability to reduce ubiquinone suggest an important antioxidant function although the effect of a null polymorphism in NQO1 on ubiquinone levels and metabolism remains to be elucidated. FAD plays an important role in stabilizing NQO1 and more generally, other proteins in the flavoproteome. Interestingly, the ability of NQO1 to bind to proteins and RNA is dependent on pyridine nucleotide redox ratio suggesting it may function as a redox-dependent molecular switch. As more data is collected on the influence of NQO1 on biological processes we will begin to understand the essential role NQO1 and similar redox-responsive enzymes play in cellular homeostasis.

## Author contributions

Both authors participated in writing this manuscript, made intellectual contributions to this work and approved it for publication.

### Conflict of interest statement

The authors declare that the research was conducted in the absence of any commercial or financial relationships that could be construed as a potential conflict of interest.
